# Social rivalry triggers visual attention in children with autism spectrum disorders

**DOI:** 10.1038/s41598-017-09745-6

**Published:** 2017-08-30

**Authors:** Marine Grandgeorge, Yentl Gautier, Pauline Brugaillères, Inès Tiercelin, Carole Jacq, Marie-Claude Lebret, Martine Hausberger

**Affiliations:** 1 Child Psychiatry Service, Centre de Ressources Autisme, CHRU of Brest, Brest, France; 2 Université de Rennes 1, UMR CNRS 6552, Ethos, Laboratoire d’éthologie animale et humaine, 35042 Rennes Cedex, France; 3 Handi’chiens Association, Alençon, France; 4 CNRS, UMR 6552 Université de Rennes 1, Ethos, laboratoire d’éthologie animale et humaine, 35042 Rennes Cedex, France

## Abstract

Visual social attention is central to social functioning and learning and may act as a reinforcer. Social rivalry, which occurs when an individual is excluded from dyadic interactions, can promote interspecific learning by triggering attention. We applied it to an animal-assisted intervention, where the behaviour of ASD children was compared between an experimental (attention shift of the animal trainer from the dog-child to the dog only) and a control (attention maintained on the dyad) groups (study 1). The results show that ASD children are sensitive to the direction of (visual) social attention and may act, physically and visually, in order to regain it. When the animal trainer concentrated on the dog, the overall visual attention of the ASD children increased, suggesting a heightened awareness towards their environment. They oriented more towards the animal trainer and the dog, contrarily to the control group. The repetition of the procedure was even associated with increased joint attention with the animal trainer (study 2). Thus, ASD children do care about and seek human visual attention. They show an ability to adapt their social behaviour, which questions whether their known deficits in social competencies are hard wired or whether the deficits are in their expression.

## Introduction

Social attention is core to social functioning and may reflect affinities^1^, and promote, the acquisition of social skills^2^. In normally developing children social attention is sought from an early age^3^ and is generally reflected through visual attention. From the first days of life, infants follow the direction of other people’s gaze, a prerequisite for joint attention which is important for language learning^4, 5^. Eye contact for children is crucial for the development of long term parent-child attachment^6^. Thus, visual attention is one major element of social functioning in establishing and maintaining communication, regulating interactions, and developing affiliative relationships^7^. Social attention may also act as a reinforcer by promoting the release of substances promoting positive emotions. For example, social bonding may increase oxytocin levels in both partners^8^, even in interspecific bonding^9^, and oxytocin is increased with visual attention in human-dog interactions^10^. Indeed, oxytocin is one likely candidate to orchestrate the emergence of the social and emotional brain in any social relationship^11^.

Social rivalry (SR) occurs when an individual is frustrated when excluded from any peer dyadic interaction^12^. SR may lead to “third party interventions” where one individual actively interferes in order to regain its partner’s social attention^12, 13^. In horses for example, positive human interactions with the mare are the best way to gain the foals attention and contact, and through such indirect interactions long term the human-foal relationships are promoted^14^.

Social modelling^15^ has been the theoretical basis for developing model-rival techniques that enhance exceptional learning^16, 17^. An example of this procedure consists of two humans working with an African grey parrot *Psittacus erithacus*, one acting as a trainer (with whom the parrot has a bond) and the other as the “rival”. The “rival”, who competes for the human’s attention by responding to the trainer’s requests, acts as a model for the bird^18^. A series of studies by Pepperberg and colleagues revealed the efficiency of this procedure in promoting speech mimicry and the referential understanding of labels^19^. In humans, observational learning has been used with children displaying autism spectrum disorders (ASD) to help them acquire appropriate lifestyle skills, e.g.^20, 21^. Outcomes may depend on the number of “models”; with a single model, (either a child or adult) present there was some imitation^22^ but when two or more models were involved there was a significant improvement for many skills, e.g. play, language; for a review see ref. 22. Pepperberg^16^ suggests that social modelling is the best way to enhance learning, and further indicates that her procedure with birds can be applied to human beings with social inhibitions or difficulties, e.g. children with hyperactive disorders or ASD. Although there is limited research on the use of the “model-rival procedure” in humans, some studies show that increased eye contact with ASD children has improved functional skill and social behaviour^17, 23^.

People with ASD are typically characterized by difficulties in communicative and social interaction skills^24^, including poor eye contact^25^ and altered processing of facial information and intentions^26^. They have atypical attentional processes^27–30^ and show a social aversion, that seems to be human-specific, avoiding looking at human faces. Indeed, they will more readily look at pictures of animals than humans^31–34^. In children with ASD, pets are a more favourable source of attention, lowering anxiety and promoting interactions^35, 36^. However, critical reports of positive outcomes are scarce and do not present definite conclusions^37^ for “animal-assisted interventions” (AAI; interventions involves animals for human benefits based on triadic interactions between a professional, a human recipient, and an animal).

In our study we adapted the model-rival procedure to an AAI triadic situation. We suggest that social rivalry may occur if the animal trainer and the service dog interacted as a dyad, concentrating their attention on each other. Because there does not appear to be social avoidance of animals in people with ASD^31, 33^, we hypothesized that this procedure, when based on a human-animal interaction, would be more effective in eliciting responses (and promoting social skills such as visual attention) towards the animal trainer^38^. Our approach differed however from the classical model-rival procedure in that there was no educational objective *per se*. We expected a spontaneous increase of social attention as a result of social rivalry because the adult’s interventions would then be indirect (hence less “invasive”). According to Richer^39^, social avoidance of humans in people with ASD may be reinforced and maintained because adults that approach, and try to interact with them independently of their responses, do so at times when the child with ASD may want to be alone. People with ASD may perceive such actions as invasive, depriving them of the role of “actor” in the interaction. In animals, studies in foals showed that indirect (e.g. via a social model) human actions were more prone to facilitate positive relationships and attention than direct actions^14, 40^.

An important limitation of most studies on AAI outcomes is the lack of control conditions (e.g. typical development group, or ASD group performed without the experimental design) and of neutral blind raters^37^. Therefore, in our first study, we compared the behaviour of children with ASD distributed in a control and an experimental group. In the control, the animal trainer performed the same usual AAI intervention (attention directed towards the child and the dog-child dyad) throughout the session. In the experimental situation, the animal trainer began as for the control, and then directed attention to the service dog only, either i) in close contact (second stage: e.g. brushing, patting), or ii) at a distance (third stage: orders to sit or stay still). In order to distinguish potential effects due to the novelty of the situation from real effects of the procedure *per se*, a further group of children with ASD was submitted to the experimental situation three times and the results compared between the first and third sessions (study 2). One concern regarding AAI is whether the child will transfer interest from the animal towards the human^41^. Joint attention, i.e. ability to coordinate attention between an object and a person in a social context^7^, as well as other communicative skills, are one major problem of people with ASD^42^ that rivalry may promote^17, 43^.

The sessions were video-recorded with one individual collecting data and two separate individuals rating the children’s behaviour. Emphasis was made on visual attention, since eye contact is strongly correlated with affiliative needs in humans^44^ and animals^45^. Gaze duration is a useful tool for evaluating individual sensitivity to visual attention^44^. If, as hypothesized, the situation triggered attention in the children with ASD, we would expect an increase in (positive) arousal state through social and non-social visual behaviours. Finally, in order to validate “social rivalry”, we recorded “third party interventions”^13^ by the children in the service dog-animal trainer dyad (i.e. physical contact such as touching the service dog or animal trainer, moving between the service dog and the animal trainer).

## Results

### Study 1

Study 1 aimed at comparing the behaviour of children with ASD in a control group or an experimental group using “social rivalry”. Children with ASD faced three successive situations corresponding to different types of attention focus from the animal trainer during an AAI session. Thus, twenty children with ASD (10 in the experimental, 10 in the control group; mean age ± SD = 7.6 ± 1.6 years) participated in a 30 minute session divided into three 10 minute periods. For the experimental group, phase 1 (P1, t_0_-t_10min_) corresponded to a usual AAI session where the animal trainer tried to involve the child in activities with the service dog (e.g. brushing, ball play) and attention was focused on the child-service dog dyad. In phase 2 (P2, t_10_-t_20min_) and phase 3 (P3, t_20_-t_30min_) the animal trainer’s visual attention was entirely focused on the service dog (i.e. SR situations) either in a context of contact (e.g. brushing; P2) or distance (e.g. commands to stay or sit; P3) interactions. For the control group, the three phases were identical to P1 (the animal trainer remained focused on the child-service dog dyad) for the whole session. The sessions were video recorded and later analysed by two raters. The raters were considered as blind as one did the data collection and two others rated the video collected.

**Figure 1:**
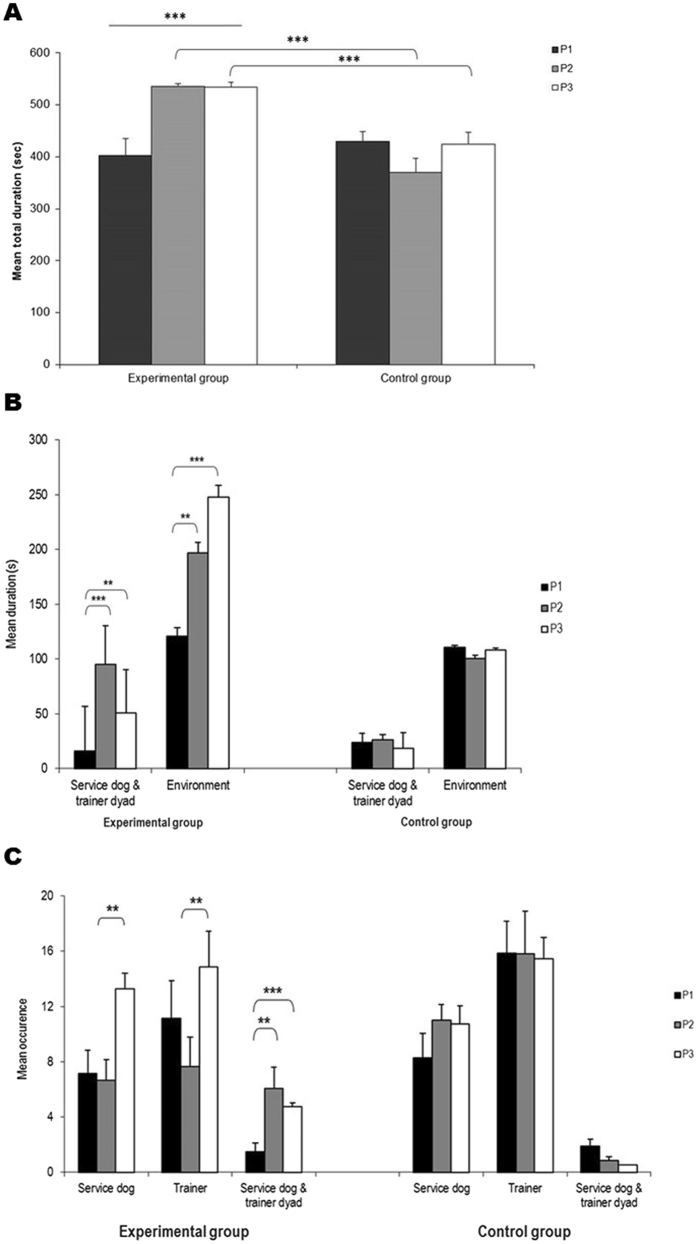
Visual attention of participants with ASD during AAI sessions. For the experimental group, animal trainers shifted their attention from the service dog-child with ASD dyad (P1) to a service dog attentional focus (P2: contact interactions; P3: distant interactions). For the control group, animal trainers focused their attention on the service dog-child with ASD dyad only. Level of significance: **p < 0.01, ***p < 0.001. (**A**) ASD participants’ overall gaze duration (in seconds). (**B**) ASD participants’ gaze duration (in seconds) according to target. (**C**) ASD participants’ glance occurrences towards the service dog and animal trainer.

The experimental group showed clear changes in visual behaviour among the three phases, with a longer gaze duration (Friedman test _(N=10, df=2)_, F = 15.44, p < 0.001; Fig. 1A) and a higher frequency of gazes (F = 5.11, p = 0.07) in phases 2 and 3 (SR situations) compared to P1. No such change was observed for the control group (Fig. 1A).

Overall, in phases P2 and P3, the experimental group showed more visual attention (i.e. time spent gazing at the animal trainer, the dog, the dyad, or the environment) than the controls (Mann Whitney U test, U = 3.67, p < 0.001; U = 3.36 p < 0.001, respectively) while the two groups did not differ in P1 (U = 0, p > 0.999).

This increase of visual attention in the experimental group in the SR situations (P2 and P3) was not specific and included both the service dog-animal trainer dyad and the environment. The pattern of visual attention also changed according to the type of interaction between the animal trainer and the service dog: more alternating glances between the service dog and the animal trainer prevailed in P3 when the service dog-animal trainer interactions were at a distance (z = 2.8 p = 0.005 and z = 2.6 p = 0.009 respectively; Fig. 1C), more focused glances towards the dyad during contact interactions (P2: z = 2.6 p = 0.009) compared to P1 (Fig. 1C).

None of these changes was observed in the control children (all Wilcoxon tests p > 0.05), who all concentrated their glances towards the trainer. During the last session, the experimental children were similar to the control children for these targets, but in addition showed a higher number of glances towards the dyad in P2 and P3 than the control group (U = 3.65, p < 0.001; U = 2.97, p = 0.002 respectively) (Fig. 1C). The experimental group therefore seemed to have an increasing awareness of the human-animal bond when the trainer’s attention was focused on the dog.

When we compared experimental and control group, no significant difference appeared for glances towards the dyad and the service dog only (all p > 0.05). The occurrence of glances towards the animal trainer was higher in the control group than the experimental group for P2 (p = 0.03).

Joint attention could not be analysed as there were too few occurrences (in total for both groups and three periods, n < 20).

**Figure 2:**
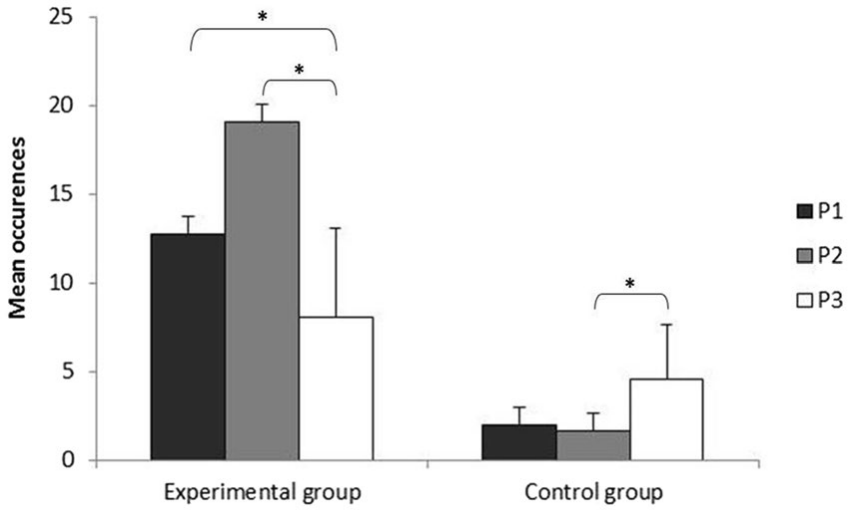
Physical interventions in the service dog and animal trainer dyad initiated by the participants with ASD during AAI sessions. For the experimental group, animal trainers shifted their attention from the service dog-child with ASD dyad (P1) to a service dog attentional focus (P2: contact interactions; P3: distance interactions). For the control group, animal trainers focused their attention on the service dog-child with ASD dyad only. Level of significance: *p < 0.05 (Wilcoxon test).

Social rivalry seemed to be involved as all children with ASD intervened physically (e.g. moving between the service dog and animal trainer, touching or grabbing the animal trainer’s arm) in the service dog - animal trainer dyad at some stage. This was especially the case for the experimental situation, where the children with ASD showed a clear increase of these behaviors in P2 (in both occurrence, z = 2.37 p = 0.018, and duration, z = 2.54 p = 0.011; Fig. 2) compared to P1. The control group showed a slight increase of these behaviours in P3 compared to P2 (occurrence, z = 1.99 p = 0.046).

To conclude study 1, children with ASD indeed proved sensitive to the focus of attention of a human trainer (and probably the service dog also) as observed when this attentional focus shifted. Competition for attention seemed to trigger their visual attention overall, but especially their social attention. This was especially true when the dyad was in close contact. Such an increase of attention along the session was not observed in the control group.

### Study 2

In the present study, 9 children with ASD (with a higher mean age of 13.7 ± 2.3 years) participated three times in the experimental SR situation. Because the most striking effects in the first study were observed by comparing P1 and P2 we then decided, for study 2, to continue the observations for shorter sessions that included only these two phases. The protocol of video recording and analysing followed that for study 1.

The results of study 2 were similar to those of study 1: the children with ASD displayed more visual attention (duration of gazes) towards the service dog - animal trainer dyad in the SR situation (P2) both in session 1 (P1 versus P2, z = 4 p = 0.02) and in session 3 (P1 versus P2, z = 4.5 p = 0.03).

**Figure 3:**
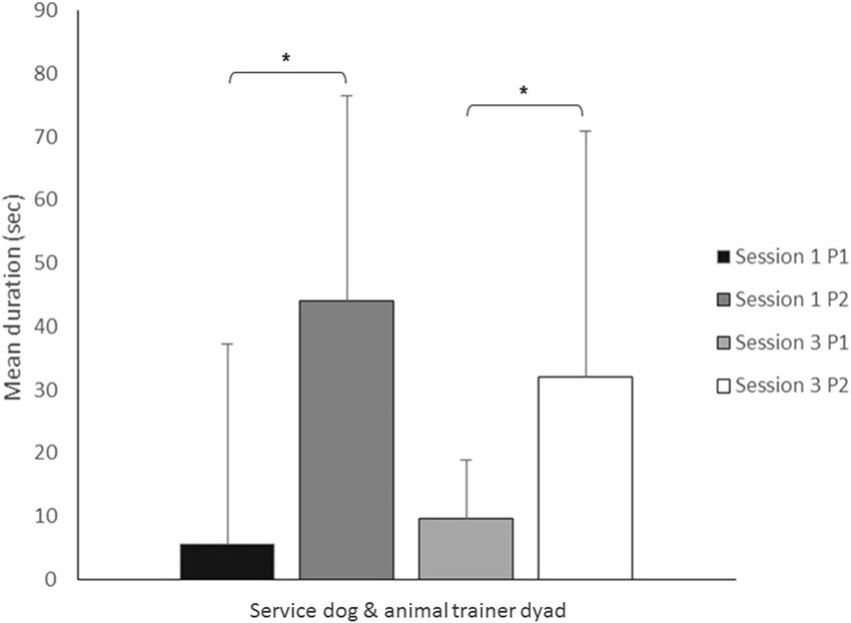
Gaze duration of children with ASD (seconds) in sessions 1 and 3. Animal trainers shifted their attention from the service dog-ASD child dyad (P1) to a service dog attentional focus (P2: contact interactions). Level of significance: *p < 0.05 (Wilcoxon test).

There was no significant change in visual behaviour of children with ASD observed between sessions S1 and S3, regardless of the phases (P1 or P2) (Wilcoxon tests, p > 0.05). In P2 of session 3, children showed a significant decrease in time gazing at the service dog while showing an increase of gazing at the dyad (Fig. 3). An increase in gaze duration toward the environment was observed in P2 of session 3 compared to P1 (z = 5.0 p = 0.044).

**Figure 4:**
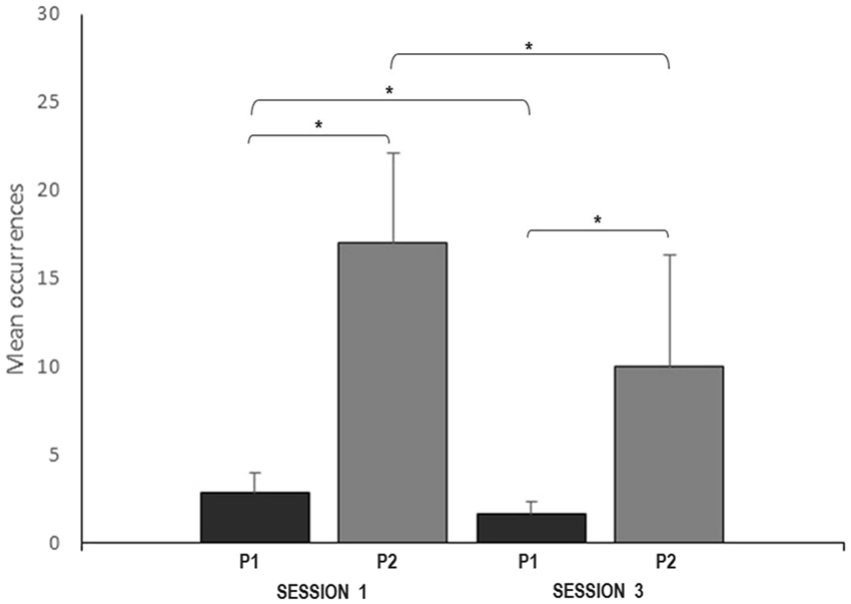
Number of occurrences of joint attention displayed by children with ASD and the animal trainer in sessions 1 and 3: Level of significance: *p < 0.05 (Wilcoxon tests).

The most remarkable result though was the striking increase (5 times) of both the occurrence and duration of joint attention between children with ASD and the animal trainer in the SR situation (P2) compared to the usual situation P1 (sessions 1 and 3; all Wilcoxon tests, p < 0.05; Fig. 4). A slight decrease on P2 was observed between session 1 and session 3 (all Wilcoxon tests p < 0.05; Fig. 4). Joint attention event between the children with ASD and the animal trainer involved mostly the service dog (85%) or service dog’s objects (8%) (e.g. ball).

To conclude this study 2, the repetition of the procedure showed maintained increased visual attention of children with ASD towards the service dog- animal trainer dyad when facing a SR situation. In this group of 13 year old children, it clearly also triggered joint attention with the human adult.

## Discussion

Our results in two different studies using a “social rivalry” procedure with children with ASD show that they are indeed sensitive to the direction of (visual) social attention, and that they act physically and visually in order to regain attention. In situations where the animal trainer concentrated on the service dog, the overall visual attention of the children with ASD increased, suggesting a heightened awareness and interactive responsiveness towards their living and non-living environment. They also clearly oriented more towards the animal trainer and the service dog, which was never observed in the control group that lacked a “social rivalry” treatment. This was especially apparent in study 2 where the children with ASD were older, and where the “social rivalry” situation triggered joint attention to the human trainer. In this study, the repetition of the AAI sessions revealed that the effect was maintained and that these major behavioural changes did not merely result from a novelty effect.

This, to our knowledge, is the first time that a potential procedure is proposed to enhance further the effects of AAI sessions on the development of social skills in children with ASD. This is also an original demonstration that children with ASD care about and seek human visual attention. Children with ASD displayed behaviors typical of other studies on “social rivalry” that demonstrate consistency in attempting to interrupt ongoing dyadic interaction and attracting attention^12, 13^. Their intervention in the service dog - animal trainer dyad suggests that children with ASD display empathy (i.e. the ability to understand and share the feeling of another) in that they recognize the bonding between the two participants (hence their “social feelings”) and attempt to participate. Although the results obtained in terms of direction of attention could for some part be explained by imitation of the visual behaviour of the animal trainer, such interventions, as well as the observation of an enhanced visual attention also towards the animal trainer, show that it is not likely to be the primary explanation for these results. This shows that children with ASD can adapt their social behaviour.

In the model-rival procedures developed by Pepperberg & Sherman^17, 23^, all case studies described mentioned an increase of eye contact. This may have been enhanced here because animals are both a source of interest^46^ and do not elicit social aversion^47^. The situation of model rivalry may well thus reveal unexpected abilities of children with ASD that are otherwise not expressed because of social inhibitions. Previously, Tinbergen & Tinbergen^48^ suggest that interaction with children with ASD should be done with minimal intrusiveness, e.g. approach with little looking. In the usual experimental situations, children with ASD tend to avoid visual contact with human faces, but not animal faces^31, 34^. In our study we found that children with ASD showed alternating gazes towards the human and service dog when both were not within their visual field. They also performed gaze following and joint attention with the animal trainer. This seems to reveal a high motivation for social attention, and in particular visual attention.

In humans during typical development, social bonding and visual attention promote the production of oxytocin^49^, even in the case of interspecific interactions^10^. People with ASD show a deficit in oxytocin levels, but administration of this hormone appears to increase their level of social interactions^50^. It may be that children with ASD, like other human beings, can benefit from this positive effect of oxytocin. However, this presumed effect may be counterbalanced by their potentially higher emotional levels that children with ASD face in complex social situations^51, 52^. Further work therefore needs to explore the interplay between oxytocin levels and visual attention in children with ASD, specifically in the context of AAI and social rivalry. As we and others have shown, the presence of an animal seems to decrease the arousal induced by direct gaze^36^ and the context of an interaction does influence its valence^53^. In the context of our study visual attention from the service dog and from the animal trainer were reinforcing and their redirection was perceived as a loss, and a potential source of frustration. Condoret^41^ observed in his anecdotal report that the “social” interest developed by a child with ASD towards a dog was extended to humans. One further reason why this “social rivalry” situation was efficient in triggering social visual attention may be because the child could choose, rather than being told, how to interact; a major issue for both humans^39^ and animals^40^ where becoming an “actor” in a relationship is important.

In conclusion, our findings raise two important points regarding the social skills of children with ASD. First, the question of whether their social competencies *per se* demonstrate hard wired deficits or whether their expression is inhibited: here the children with ASD revealed their sensitivity to social attention across a broad child age range and their ability to modulate their behaviour accordingly. These findings suggest a novel vision of the ASD social deficits and the mechanisms involved that should promote the development of further studies in more natural settings and with larger samples. Second, this research suggests the importance of examining the procedures of AAI, especially its potential role as an alternative or complementary approach for recovery.

## Material and Methods

Data were collected in April-May 2013 and February-March 2014.

### Ethical concern

Regarding dogs, the study was conducted in accordance with the French regulations governing the care and use of research animals. Regarding humans, all parents provided free and informed consent for the participation of their child in the study. All human-related methods were performed in accordance with the Declaration of Helsinki (6^th^ revision), and French regulations. All procedures were approved by the regional ethical committee (*CPP Ouest V*).

### General study protocol

Thirty one participants were recruited from the service dog adoption waiting list of the Handi’chiens association as well as from 3 French institutions specialized in ASD. We ensured that all^1^, had been diagnosed with ASD according to DSM-IV^54^ (for details of recruitment and eligibility: SI Materials and Methods)^2^, were aged 4 to 18 years^3^, had no prior parent-reported history of animal abuse, and^4^ had no physical disability that could limit their interactions with the service dog.

The other individuals included in the research were five professional animal trainers (who had received the same training in Handi’chiens association) and two program facilitators. Finally, 11 service dogs (2 females, 9 males; all aged 22 to 25 months) were included. They had all received the same training^55^ by same animal trainers (standardised method) and were able to respond to approximately fifty commands (e.g. stay calm, lying, turn on the light). Two dog breeds were used, i.e. labrador retriever and golden retriever (for details about the different participants: SI Materials and Methods)

### Participants and experimental design

During each experimental session, the participant with ASD, the program facilitator, the service dog, the ASD familiar carer (either parent or institutional carer) were always present and received standardised instructions (for details: SI Materials and Methods).

#### Study 1

*Target population with ASD*. The participants were 20 individuals with ASD (mean age ± SD = 7.6 ± 1.6 years) assigned randomly to one of two groups: 10 (9 males, 1 female) to an experimental group and 10 (9 males, 1 female) to the control group.

*Experimental group procedure*. The session lasted 30 minutes divided into three periods of 10 minutes. Period 1 (t_0_-t_10_) was the baseline for the experiment consisting of “free time”, i.e. the animal trainer acted as usual in his/her AAI session, mostly trying to actively involve the child in physical interactions with the service dog, e.g. asking him/her to brush the service dog. This period was considered as the control situation for each session. In the SR situations, the animal trainer focused his/her attention on the service dog; in Phase 2 (t_10_-t_20_) having direct contact with the service dog and in Phase 3 (t_20_-t_30_) only distant interactions (at a distance of > 1 m).

*Control group procedure*. The session consisted of a single period of 30 minutes “free time”, identical to the P1 situation of the experimental group. For the analyses, the 30 minutes were divided into 3 phases of 10 minutes to be compared to experimental group data.

#### Study 2

*Target population with ASD*. The participants were 9 individuals with ASD (8 males, 1 female; mean age ± SD = 13.7 ± 2.3 years).

*Procedure*. All experiments were performed at the institutions of participant with ASD (*i*.*e*. in a familiar environment). Each week, an appointment was made (20 minutes for each participant with ASD). Each participant with ASD was his/her own control. Based on the results of study 1, we followed up with P1 (t_0_-t_10_) and P2 (t_10_-t_20_) 20 minutes of experiment in 3 sessions at one week intervals. According to institutional constraints, the real time between two sessions was 5.9 ± 1.9 days.

### Data analyses

All AAI sessions were video recorded for later analysis.

#### Behavioral measurements

In study 1, all video recordings from all sessions were analysed. In study 2, we focused on the first and the last sessions for each participant with ASD. Recordings were analysed using a focal sampling method: all behaviors of the focal participant with ASD were coded continuously using the software The Observer 11.5 version^56^ and were expressed in occurrence and in duration (seconds).

Visual attention was measured as gazes and glances (less than one second^57^ and the target was defined as being within 5° of the eye orientation^7^. The target could be: (a) the service dog, (b) the animal trainer, (c) the service dog and animal trainer dyad, (d) the environment including objects or the program facilitator in the experimental room. If the target was not clearly identified, it was recorded as non-visible. Occurrences of joint attention were also recorded^7^. Only instances of joint attention initiated by visual cues was recorded (i.e. we excluded vocal and tactile initiation).

Any spontaneous interventions by the participant with ASD in the animal trainer-service dog dyad were recorded: they consisted of behaviours aimed clearly at interrupting the ongoing dyadic interaction and attracting attention (i.e. physical contact such as touching the service dog or animal trainer, moving in between the service dog and the animal trainer)^58^.

#### Statistical analyses

As data were not normally distributed, we used non-parametric statistical tests^59^. The Friedman test and Wilcoxon signed rank t-tests were used to compare matched paired data (*i*.*e*. comparison of the behaviour of the same individual at different periods). Mann-Whitney U-tests were used to compare the two groups of participants with ASD (experimental *versus* control) in each period. To allow this comparison, sessions of the control group were divided into three equal periods of 10 minutes; each corresponds to the three periods of the experimental group (*i*.*e*. P1, P2 and P3). These analyses were run with StatView software© and R software© with an accepted p-level at 0.05.

## Electronic supplementary material

Supplementary material
