# Supplementary material for: Social rivalry triggers visual attention in children with autism spectrum disorders
Source: Sci Rep. 2017 Aug 30;7:10029. doi: 10.1038/s41598-017-09745-6 (PMC5577136; doi:10.1038/s41598-017-09745-6)
Supplement: Supplementary file 1 — Supplementary material [file 41598_2017_9745_MOESM1_ESM.docx]

**Social rivalry triggers visual attention in children with autism spectrum disorders**

Marine Grandgeorge ^a,b*^, Yentl Gautier ^b^, Pauline Brugaillères ^b^, Inès Tiercelin ^b^, Carole Jacq ^b^, Marie-Claude Lebret^c^, Martine Hausberger ^d^

^a^ Child Psychiatry Service, *Centre de Ressources Autisme*, CHRU of Brest, Brest, France

^b^ Université de Rennes 1, UMR CNRS 6552, Ethos, Laboratoire d’éthologie animale et humaine, 35042 Rennes Cedex, France

^c^ Handi’chiens Association, Alençon, France

^d^ CNRS, UMR 6552 Université de Rennes 1, Ethos, laboratoire d’éthologie animale et humaine, 35042 Rennes Cedex, France

**Supplementary information**

**Materials and Methods**

**1. Recruitment and eligibility**

***Criteria.*** Inclusion criteria for target participants with ASD included (a) age between 4 to 18 years, (b) a parent-reported diagnosis of ASD and a medical report of ASD using ADI-R^1^ and made according to DSM-IV ^2^, and (c) no prior parent-reported history of animal abuse. Alternatively, in the absence of a medical report of ASD, parents completed the Social Communication Questionnaire (SCQ) ^3^ and we included participants with a minimum score of 15 that indicates the presence of ASD. Exclusion criteria included (a) physical disability that could modify the interactions with the service dog (e.g. wheelchair, blindness), (b) a score of 14 or less in the SCQ, and (c) participants who did not complete the whole experimental procedure.

***Social Communication Questionnaire (SCQ).*** The SCQ is a 40-item parent-report screening questionnaire for ASD ^3^. We used the SCQ French version ^4^. Items on the SCQ correspond to criteria used to diagnose the core features of ASD (*i.e.* communication, reciprocal social interactions, and repetitive behaviours and interests). This autism screening questionnaire is the best researched and validated parent-report screening tool for ASD ^5^ as it shows strong discrimination between ASD and non-ASD cases as well as good reliability and validity ^6^. The lifetime version was used in the current study. Each item on the SCQ is rated as ‘‘yes’’ or ‘‘no’’ and assigned a 0–1 point rating (0 = absence of abnormal behaviour, 1 = presence of abnormal behaviour). Items address both current and past behaviour. The possible range of scores for nonverbal children is 0–33 and for verbal children is 0–39. In the French version, the cut-off score used for ASD screening purposes is 15 ^4^.

***Participant selection.*** We contacted the families of 38 eligible participants, selected from the dog adoption waiting list of Handi’chiens association ([www.handichiens.org](http://www.handichiens.org/)) and from the individuals belonging to the 3 ASD institutions. The parents received full information about the study. From the initial pool of individuals, 2 families declined the invitation; the others gave their consent. Twelve families provided a medical report with an ASD diagnosis using ADI-R, made according to DSM-IV. Twenty four parents completed the SCQ, 6 of them had a SCQ score below 15 and 1 did not follow the whole protocol of the study 2.

***Service dog selection.*** All service dogs were provided by the Handi’chiens association where they received appropriate training and behavioural evaluation. The service dog training lasts 22 months and is divided into several steps, before being adopted by an individual or a family. Here, in the case of service dogs trained for individuals with ASD, the main goal of the training is to teach service dogs to remain calm in order to ensure safety should their environment become chaotic. Selected service dogs were deemed to adapt easily to various environments, to be even-tempered, to be calm and respectful, in an attempt to increase the family’s quality of life. The current study involved service dogs who had completed their training, just before their adoption. As all the service dogs received the same training, individual variation between them is quite limited.

**2. The target population**

***2.1. Study 1***

***Participants with ASD.*** Twenty participants were included (18 males, 2 females) aged 4.9 to 14.1 years (mean age ± SD=7.6±1.6). Ten participants had received a medical diagnosis of ASD. The other parents (n=10) had completed the SCQ with a mean score of 26.0±3.8. The sample of participants was separated randomly into two groups. An experimental group included 10 participants with ASD (9 males, 1 female) with a mean age of 6.8±2.7 years old. The control group included 10 participants with ASD (9 males, 1 female) with a mean age of 8.4 ± 3.7 years old. They were matched for chronological age and gender (both p>0.05).

***Animal sample.*** Nine service dogs were included: 3 Labrador retrievers, 6 golden retrievers (8 males, 1 female; mean age±SD: 23.8±0.5 months).

***Animal trainers.*** Three different animal trainers (1 male and 2 females; mean age±SD: 44.3±7.7) of Handi'Chiens association participated in the study. They were professional dog handlers and had extensive experience of animal assisted interventions, working with individuals with ASD. They received specific instruction in our experimental procedure from one of the authors (MG).

***Program facilitator.*** All sessions took place under the supervision of the program facilitator, one of the authors (IT or CJ, female, 23 years old). Prior to the session, the facilitator met the family and the participants with ASD to introduce the different stages of the research. During sessions, the program facilitator was present to video record the session.

***3.2. Study 2***

***Participants with ASD.*** The nine participants with ASD were aged between 11.1 and 18.0 years. Two participants had received a medical diagnosis of ASD. The other parents (n=7) had completed the SCQ with a mean score of 27.3±2.6.

***Animal sample.*** Two service dogs were included: 1 male Labrador retriever, 1 female golden retriever (mean age±SD: 23.9±0.2 months).

***Animal trainers.*** Two different animal trainers (1 male and 1 female; mean age±SD: 34.5±1.1) of Handi'Chiens association participated of the current study. One was a professional dog handler with experience of animal assisted interventions, working with individuals with ASD. The other was a social worker specialized in ASD and who has worked as a volunteer in Handi'Chiens association for 10 years, familiar with the specific dog training system. They both received specific instruction in our experimental procedure from one of the researchers (MG).

***Program facilitator.*** All sessions took place under the supervision of the program facilitator, one of the authors (YG, female, 25 years old). Prior to the session, she had a special meeting with the family (by phone or in person), and met the institution team (especially the institution carer) and the participants with ASD to introduce the different stages of the research. During sessions, the program facilitator was present to video record the session.

**3. Experimental design**

***3.1. General instructions***

Each experimental room was quite similar in size (around 20 meters square), with a low table in the middle and dog-related objects (e.g. balls, leash, brush, dog toys).

Before setting up the experiment, the program facilitator instructed the participants and their familiar carers about her role during the experiment as program facilitator and their own roles:

- The participant with ASD could behave as he/she chose. For example, he/she was free to interact with the service dog and the animal trainer. We stressed to the participant with ASD and the familiar carers that no behaviour was considered either right or wrong.
- The familiar carers were asked to sit in a corner of the room, to stay neutral and silent (*e.g.* no encouragement to the participant with ASD).

After ensuring that the instructions had been understood, the program facilitator asked the familiar carers to sit on chairs in one corner, the participant with ASD to stay in the middle of the room. Then the animal trainer and the service dog entered the room. As soon as they entered, the program facilitator switched on her camera. The program facilitator remained neutral and silent in an unobtrusive place in the room; she moved only if absolutely necessary to avoid losing a frontal view of the participant with ASD (*e.g.* participant with his/her back to the program facilitator) and stopped the experiment at the end of the session.

***3.2. General procedure***

*Study 1*

*Experimental context.* An appointment of two hours was set at least 2 weeks before the experiment. All experiments were performed at Handi’chiens center, Alençon, France. At the time of the appointment and for at least one hour, individuals with ASD were free to play in the experimental room to avoid any anxiety because of unfamiliar surroundings.

*Experimental group procedure*. The session lasted 30 minutes divided into three periods of 10 minutes. During period 1 (P1: t_0_-t_10_) the animal trainer mainly tried to get the participant with ASD to interact with the service dog, and vice versa. During period 2 (P2: t_10_-t_20_), the animal trainer carried out activities in contact with the service dog, always in the same order in each session. The animal trainer (1) patted the service dog, (2) brushed its fur, (3) examined its legs and its ears, (4) brushed the service dog’s teeth, (5) put on and removed its collar, and (6) whispered in its ear. During period 3 (P3: t_20_-t_30_), the animal trainer began by moving away from the low table where the service dog was sitting. Then, the animal trainer initiated distant interactions with the service dog, always in the same order in each session. The animal trainer (1) gave commands (*e.g.* sit, down), and asked the service dog to (2) bring back a ball, (3) bark, (4) lie down under the low table, (5) get onto the low table, (6) get down from the low table and (7) bring an object. During P3, the service dog could get down from the low table and move around the room. If the participant tried to communicate with the animal trainer in P2 or P3 (*e.g.* verbal solicitation, physical contact), the animal trainer responded verbally but did not follow up the interaction and did not encourage the participant. If the participant with ASD was interacting with the service dog, the animal trainer continued to behave in the same way (*i.e.* her/his attention directed towards the service dog) and let the ASD participant interact with the service dog.

*Control group procedure.* The session consisted of a single period of 30 minutes free time, *i.e.* baseline for the experimental group (P1).

*Study 2*

*Experimental context.* Each week, an appointment was made; the first appointment was set at least 2 weeks before the experiment. All experiments were performed in 3 French institutions specialized in ASD. At the time of the appointment, here again, individuals with ASD were free to play in the experimental room but time was reduced as it was a familiar environment.

*Experimental procedure*. Three sessions, one per week, were performed. The session lasted 20 minutes divided into two periods of 10 minutes. During period 1 (P1: t_0_-t_10_) the animal trainer mainly tried to get the participant with ASD to interact with the service dog, and vice versa. During period 2 (P2: t_10_-t_20_), the animal trainer carried out activities in contact with the service dog, always in the same order in each session (see study 1 for details). Here again, if the participant tried to communicate with the animal trainer or the service in P2 (*e.g.* verbal solicitation, physical contact), the animal trainer behaved as described in study 1.

**References**

1. Lord C, Rutter M, Le Couteur A. Autism Diagnostic Interview-Revised: a revised version of a diagnostic interview for caregivers of individuals with possible pervasive developmental disorders. *Journal of Autism and Developmental Disorders.* 1994;24(5):659-685.

2. American Psychiatric Association. *Diagnostic and Statistical Manual of Mental Disorders.* Washington1994.

3. Rutter ML, Bailey A, Lord C. *Social Communication Questionnaire.* Los Angeles: Western Psychological Services; 2003.

4. Kruck J, Baduel S, Rogé B. *SCQ: Questionnaire de Communication Sociale pour le Dépistage des Troubles du Spectre Autistique.* Paris: Hogrefe; 2013.

5. Norris M, Lecavalier L. Screening accuracy of level 2 autism spectrum disorder rating scales: A review of selected instruments. *Autism.* 2010;14:263-284.

6. Witwer A, Lecavalier L. Autism screening tools: An evaluation of the social communication questionnaire and the developmental behaviour checklist autism screening algorithm. *Journal of Intellectual and Developmental Disability.* 2007;32:169-179.
